# Characterization of a *Bacillus subtilis* S-16 Thiazole-Synthesis-Related Gene *thiS* Knockout and Antimicrobial Activity Analysis

**DOI:** 10.3390/cimb45060292

**Published:** 2023-05-26

**Authors:** Jinghan Hu, Zhenhe Su, Baozhu Dong, Dong Wang, Xiaomeng Liu, Huanwen Meng, Qinggang Guo, Hongyou Zhou

**Affiliations:** 1 College of Horticulture and Plant Protection, Inner Mongolia Agricultural University, Hohhot 010020, China; 2 Institute of Plant Protection, Hebei Academy of Agricultural and Forestry Sciences, Baoding 071000, China; 3 Inner Mongolia Cold and Arid Region Crop Protection Engineering Technology Center, Hohhot 010020, China

**Keywords:** 2-methyl benzothiazole, *Bacillus subtilis*, *thiS*, volatile organic compound, *Sclerotinia* wilt of sunflower

## Abstract

*Bacillus subtilis* S-16 isolated from sunflower-rhizosphere soil is an effective biocontrol agent for preventing soilborne diseases in plants. Previous research revealed that the volatile organic compounds (VOCs) produced by the S-16 strain have strong inhibitory effects on *Sclerotinia sclerotiorum*. The identification of the VOCs of S-16 using gas chromatography-tandem mass spectrometry (GC-MS/MS) revealed 35 compounds. Technical-grade formulations of four of these compounds were chosen for further study: 2-pentadecanone, 6,10,14-trimethyl-2-octanone, 2-methyl benzothiazole (2-MBTH), and heptadecane. The major constituent, 2-MBTH, plays an important role in the antifungal activity of the VOCs of S-16 against the growth of *Sclerotinia sclerotiorum*. The purpose of this study was to determine the impact of the *thiS* gene’s deletion on the 2-MBTH production and to conduct an antimicrobial activity analysis of the *Bacillus subtilis* S-16. The thiazole-biosynthesis gene was deleted via homologous recombination, after which the contents of 2-MBTH in the wild-type and mutant S-16 strains were analyzed using GC-MS. The antifungal effects of the VOCs were determined using a dual-culture technique. The morphological characteristics of the *Sclerotinia sclerotiorum* mycelia were examined via scanning-electron microscopy (SEM). Additionally, the lesion areas on the sunflower leaves with and without treatment with the VOCs from the wild-type and mutant strains were measured to explore the effects of the VOCs on the virulence of the *Sclerotinia sclerotiorum*. Moreover, the effects of the VOCs on the sclerotial production were assessed. We showed that the mutant strain produced less 2-MBTH. The ability of the VOCs produced by the mutant strain to inhibit the growth of the mycelia was also reduced. The SEM observation showed that the VOCs released by the mutant strain also caused more flaccid and gapped hyphae in the *Sclerotinia sclerotiorum*. The *Sclerotinia sclerotiorum* treated by the VOCs produced by the mutant strains caused more damage to the leaves than that treated by the VOCs produced by the wild type and the mutant-strain-produced VOCs inhibited sclerotia formation less. The production of 2-MBTH and its antimicrobial activities were adversely affected to varying degrees by the deletion of *thiS*.

## 1. Introduction

*Sclerotinia* wilt in sunflowers caused by *Sclerotinia sclerotiorum* is one of the main airborne and soilborne diseases of sunflowers, and it causes substantial global economic losses [1]. Sclerotia, which are the main resting structures of *S. sclerotiorum* resting in the soil [2], germinate under suitable conditions to produce asci in the following year. Mature asci release many ascospores, which are dispersed by airflow or rain and subsequently germinate to produce mycelia that can infect sunflower plants [3]. Because the sclerotia remain viable in the soil for long periods and there is currently a lack of resistant sunflower varieties, crop rotation and the cultivation of disease-resistant varieties are relatively ineffective for controlling *Sclerotinia* wilt in sunflowers [4]. Although chemical fungicides have been used to control *Sclerotinia* in sunflowers, the excessive application of these chemicals results in polluted environments and has detrimental effects on soil ecology [5]. Therefore, there is an urgent need for the development of alternative methods for suppressing *Sclerotinia* in sunflowers. Biological control measures involving microbes have been confirmed as efficient and environmentally friendly methods for s-suppressing soilborne diseases in plants [6].

*Bacillus subtilis* and its close relatives are important resources for the development of microbial fungicides, partly because they produce stress-resistant spores that may be incorporated into products with long shelflives. Previous research revealed that *B. subtilis* suppresses soilborne diseases by producing antifungal compounds that induce systemic resistance in plants, and by competing with phytopathogens for nutrients and niches [7]. The production of antifungal compounds is a common characteristic among biocontrol agents, including *B. subtilis* [8,9]. Lipopeptide antibiotics are the main antifungal compounds produced by *B. subtilis* [10,11]; however, the volatile organic compounds (VOCs) produced by some biocontrol strains of *B. subtilis* also have strong inhibitory effects on phytopathogens, making them important for protecting plants from soil-borne diseases [12]. More specifically, VOCs disrupt the growth of phytopathogen growth, either directly or through microbial interactions [13]. Additionally, volatile compounds can diffuse easily in the air contained in soil pores, enabling them to disperse farther in soil than bacterial soluble compounds [14]. Bacterial VOCs have antimicrobial activities and can increase plant growth, interact with the host, and function as signaling molecules in bacterial communities. For example, n-decanal inhibits the growth of *S. sclerotiorum* growth [5], allyl alcohol positively affects beneficial bacterial populations, and 2,3-butadienol increases plant growth [15]. Phenol is a useful antiseptic in clinical applications because of its cytotoxic properties [16]. Some studies revealed the antifungal activities of certain volatile compounds produced by antagonistic fungi and bacteria [5,17,18]. Moreover, the utility of antifungal volatile compounds from fungal strains for the biocontrol of plant diseases was investigated under greenhouse conditions [19,20]. However, these earlier studies were insufficient for exploiting the diverse microorganisms in the soil. There are relatively few reports describing the precise repressive effects of antifungal compounds on soilborne plant diseases.

The *B. subtilis* strain S-16 can suppress *Sclerotinia* wilt in sunflowers because its VOCs inhibit mycelial growth and the formation of sclerotia by *S. sclerotiorum* fungi [21]. A previous study demonstrated that 2-methyl benzothiazole (2-MBTH), which is the main component in the volatile compounds produced by this strain, has strong inhibitory effects on the mycelial growth and sclerotial germination of *S. sclerotiorum* fungi [22]. Additionally, 2-MBTH is a derivative of benzothiazole, which has promising inhibitory activities against *S. sclerotiorum* [5,23,24]. The whole genome of *Bacillus subtilis* S-16 was analyzed (GenBank: CP063150.1), and the *thiS* gene was identified in the genome and functionally annotated as a thiazole-synthesis-related gene. The objective of this study was to determine the impact of the *thiS* gene’s deletion on the 2-MBTH production and to conduct an antimicrobial activity analysis of the *B. subtilis* S-16.

## 2. Materials and Methods

### 2.1. Bacterial Strains, Fungal Pathogens, and Growth Conditions

The bacterial strains and plasmids used in this study are listed in Table 1. The *B. subtilis* strain S-16 was isolated from the soil surrounding sunflower roots in Salazi, Baotou, Inner Mongolia [25]. The *B. subtilis* strains were stored at −80 °C in Luria–Bertani (LB) broth containing 30% glycerol. Fresh bacterial cultures were routinely retrieved from frozen stocks before each experiment and grown at 37 °C on LB agar medium. The *Escherichia coli* DH5α cells used as the hosts for constructing recombinant plasmids were cultured at 37 °C in LB medium. The X-8 strain used for testing was collected from diseased sunflower plants in Wuyuan County, Bayannur, Inner Mongolia, by staff from the Laboratory of Molecular Plant Pathology of Inner Mongolia Agricultural University. After surface disinfection and purification on PDA medium, the strain was identified as *S. sclerotiorum* via sequencing [26]. The strain was maintained on PDA slants, which were supplemented with 20% glycerol and kept at −80 °C for long-term storage.

### 2.2. Strain Construction

The analysis of the genome sequence of *B. subtilis* S-16 (GenBank: CP063150) revealed that the *thiS* gene encodes a sulfur-carrier protein (protein ID: BSn5_17755). To functionally characterize this gene in terms of its effects on the production of 2-MBTH and its role in inhibiting the growth of *S. sclerotiorum*, a mutant S-16 strain was produced with an in-frame deletion of *thiS*. Specifically, the temperature-sensitive vector pKSV7 [27] was used to remove an internal fragment of the *thiS* gene in strain S-16. The deletion of *thiS* in the S-16 strain was performed as previously described using homologous recombination [28]. Specifically, the upstream region was amplified by PCR using the primer pair *thiS*-1 and *thiS*-2, whereas the downstream region was amplified by PCR using the primer pair *thiS*-3 and *thiS*-4. The resulting PCR products were linked using the primer pair *thiS*-1 and *thiS*-4, which included the *Eco*RI and *Bam*HI restriction sites, respectively. This PCR fragment was introduced into pKSV7, and then the recombinant plasmid was inserted into strain S-16 via electroporation. The knockout mutant was confirmed by PCR amplification and sequencing using the primer pair *thiS*-1 and *thiS-*4. To produce a complementary strain, a fragment containing the intact *thiS* gene and the 500-bp upstream sequence was amplified from *B. subtilis* S-16 chromosomal DNA using the primer pair *thiS*-comF (with the *Bam*HI restriction site) and *thiS*-comR (with the *Hin*dIII restriction site). The PCR product was digested using *Eco*RI and *Hin*dIII, and cloned into the *EcoR*I and *Hin*dIII sites of pHY300PLK [29]. The recombinant plasmid was introduced into the mutant via electroporation.

### 2.3. Measurements of the Growth Curve

To analyze the growth patterns of the wild-type strain S-16, as well as the *thiS* deletion mutant and its complementary strain, the *B. subtilis* strains were grown overnight at 37 °C in LB medium. The overnight cultures were used to inoculate fresh LB medium at an optical density at 600 nm (OD_600_) of 1. The *B. subtilis* strains were cultured at 37 °C with shaking (180 rpm). The growth of the *B. subtilis* strains was monitored by measuring the OD_600_ at 3-h intervals for 36 h. This experiment was completed using three biological replicates. The growth curves were plotted on the basis of the mean values and standard deviations.

### 2.4. In Vitro Antifungal Activities of the VOCs Produced by B. subtilis S-16 and Its Mutants

Cultures of *Bacillus subtilis* S-16 and its mutants were used to inoculate 5 mL of LB broth and grown overnight. An aliquot (1%) of the overnight culture was used to inoculate 50 mL of fresh LB broth, which was then incubated at 30 °C with shaking (180 rpm) for 12 h. Next, 200 µL samples of *B. subtilis* S-16 and its mutants were transferred to one compartment of a divided Petri dish and spread evenly on the surface of the LB agar medium. The other compartment containing PDA medium was inoculated by placing a *S. sclerotiorum* disc (5-mm diameter) at the center. Furthermore, LB agar plates without *B. subtilis* S-16 or its mutants were used as controls. The Petri dishes were sealed using parafilm and incubated at 25 °C. After 4–7 days, the diameters of the fungal colonies were measured and the rate of mycelial growth inhibition was determined. After 7 days of incubation at 20 °C, the growth of the sclerotia was evaluated visually. The number of sclerotia per treatment was recorded and the weight of the sclerotia was measured.

### 2.5. Scanning Electron Microscopy

The morphological characteristics of the mycelia of *S. sclerotiorum* treated with the VOCs from the wild-type and mutant *B. subtilis* S-16 strains were visualized using scanning-electron microscopy. Briefly, *S. sclerotiorum* was co-cultured with the wild-type and mutant *B. subtilis* S-16 strains for 7 days at 25 °C using divided Petri dishes. The treated mycelia were collected and fixed in 2% glutaraldehyde at 4 °C and then dehydrated using an ethanol gradient (30%, 50%, 80%, 90%, and 100%). The mycelia were lyophilized, coated with gold, and examined using an S-3500N field-emission scanning-electron microscope (Hitachi, Tokyo, Japan). The experiment was repeated three times.

### 2.6. In Vivo Antifungal Activities of the VOCs Produced by B. subtilis S-16 and Its Mutants

The utility of the VOCs from the wild-type S-16, the mutant strain Δ*thiS*, and the complementary strain Δ*thiS* for the in vivo control of *S. sclerotiorum* was evaluated using detached sunflower leaves. The wild-type and mutant *B. subtilis* S-16 strains were cultured overnight in LB broth at 30 °C with shaking (180 rpm). A 200 µL aliquot of each overnight culture was transferred to one compartment of a divided Petri dish and spread evenly over the surface of the LB agar medium. Fresh sunflower leaves (LD 5009) were collected and gently rinsed with sterile water. After air-drying on a clean bench, the leaves were inoculated with a 6-mm agar plug containing *S. sclerotiorum* and then placed on wet filter paper in the other compartment of the divided Petri dishes. The leaves were incubated at 25 °C under a 12 h-light: 12 h-dark photoperiod for 5 days. The lesion areas were measured using the crossover method and the inhibitory efficiency was calculated as inhibitory efficiency = (control lesion diameter–treatment lesion diameter)/control lesion diameter × 100%. The control lesion diameter represents the diameters of the lesions without the VOC treatment, treatment lesion diameter represents the diameters of lesions after treatment with VOCs produced by the wild-type S-16, the mutant strain Δ*thiS*, or the complementary strain Δ*thiS*.

### 2.7. Collection of VOCs via SPME and GC-MS Analysis

To extract the VOCs, 20 mL of LB medium in 100-mL vials was inoculated with the wild-type S-16, the mutant strain Δ*thiS*, or the complementary strain Δ*thiS*. The vials were incubated at 37 °C for 3 days and then used for the subsequent analyses. To ensure the results of the experiment were reproducible, four samples were prepared for each strain. Additionally, non-inoculated LB medium was used as the control. The volatile organic compounds were analyzed via solid-phase microextraction (SPME) coupled with gas chromatography-tandem mass spectrometry (GC-MS). A SPME fiber (65 µm divinylbenzene/carboxen/polydimethylsiloxane fiber) was inserted into the headspace of the flask, which was incubated at 37 °C for 7 h. The compounds were then desorbed for 20 min in the injection port of the gas chromatograph at 220 °C with the purge valve turned off (splitless mode). An HP-5 capillary column (30.0 m × 0.25 mm × 0.25 µm, Thermo, Waltham, MA, USA) with helium as the carrier gas was used for the GC-MS/MS analysis. A Thermo Trace 1300 ISQ MS system was used for separating and detecting the peaks. Each run was 45 min long. The initial oven temperature (40 °C) was held for 4 min. The temperature was then increased at a rate of 5 °C/min to 150 °C. After holding for 1 min, the temperature was increased at a rate of 10 °C/min to 280 °C and held for 5 min. The mass spectrometer was operated in electron-ionization mode at 70 eV with a source temperature of 280 °C for a continuous scan (35–400 *m/z*). The analysis was performed in the full-scan mode. The mass spectral data of the VOCs were compared with the data in the National Institute of Standards and Technology Mass Spectrum Library.

### 2.8. Statistical Analyses

Three independent experiments were conducted for each assay. A two-way ANOVA was performed for the statistical analyses, which were carried out using SPSS 18.0 software (SPSS, Chicago, IL, USA), with *p* ≤ 0.05 set as the threshold for determining the significance of any differences.

## 3. Results

### 3.1. Knockout of thiS from the Genome of B. subtilis S-16

**Figure 1:**
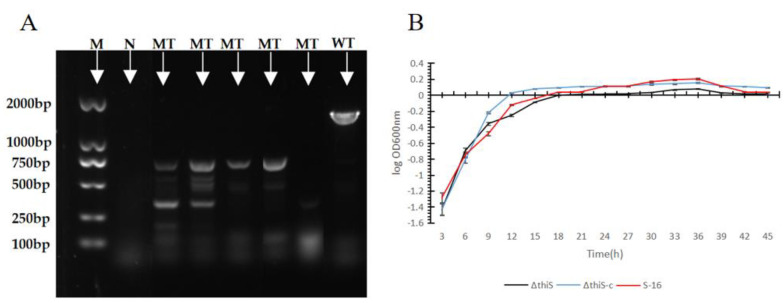
(**A**) Confirmation of the knockout of *thiS*. PCR amplification and sequencing were carried out with the primers *thiS*-1 and *thiS*-4, and the PCR products were analyzed using agarose-gel electrophoresis. Lane M (the first lane): DNA marker (this marker was purchased from Takara. The size of the tape was known and fixed. The operation was carried out according to the product instructions); Lane N (the second lane): no template control; lane WT (the eighth lane): genomic DNA of wild-type S-16 as the template; Lane MT (three-to-seven lane): mutant strains. (**B**) Growth curves of the wild-type S-16, the mutant strain Δ*thiS*, and the complementary strain Δ*thiS*-*c*.

The *thiS* gene encodes a 7310-Da sulfur carrier that participates in the thiamin-biosynthesis pathway *of B. subtilis*. The full-length gene sequence contains 203 bp. Its functions include the processing of genetic information and the folding, sorting, and degradation of proteins. To explore the impact of the *thiS* gene’s deletion on the 2-MBTH production and antimicrobial activity analysis of the *B. subtilis* S-16, the gene was deleted via in-frame mutagenesis involving the temperature-sensitive vector pKSV7. The PCR amplification and sequencing with the primers *thiS-*1 and *thiS*-4 confirmed that the *thiS* sequence was disrupted in the mutant (Table 2). Five transformants that had undergone homologous recombination were obtained and one was selected for further study (Figure 1A), which was named Δ*thiS* in this study. To generate the complementary strain, the intact gene was introduced into the Δ*thiS* mutant strain using the shuttle vector pHY300PLK. Four complementary strains were obtained and the selected complementary strain was named *ΔthiS-c*. There were no significant differences between the growth curves of the wild-type and mutant S-16 strains (Figure 1B).

### 3.2. In Vitro Antifungal Activities of the VOCs from B. subtilis S-16 and Its Mutants

**Figure 2:**
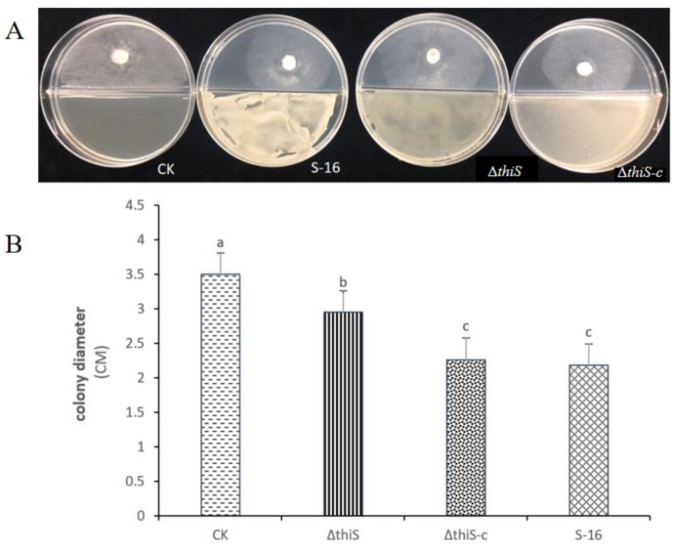
Effects of the wild-type S-16, the mutant Δ*thiS*, and the complementary strain Δ*thiS*-c on mycelial growth. (**A**) Effect of VOCs produced by the wild-type S-16, the mutant strain ∆*thiS*, and the complementary strain ∆*thiS-c* on the mycelial growth of *S. sclerotiorum*. (**B**) Mycelial growth with and without treatment with the volatile compounds of wild-type S-16, the mutant strain Δ*thiS,* and the complementary strain Δ*thiS-c*. The data are the mean ± s.d. (n = 3). Matching letters on the bars for each column indicate no significant difference according to the LSD test at *p* = 0.05.

The antifungal activities of the VOCs from the wild-type and mutant strains of S-16 were compared using divided Petri dishes. The mycelia of the *S. sclerotiorum* in the untreated control grew rapidly and covered the whole dish at 3 days post-inoculation. The mycelial growth of the *S. sclerotiorum* was significantly inhibited by the wild-type S-16 strain, but the co-culturing with the complementary strain increased the mycelial growth of *S. sclerotiorum*. Accordingly, the *thiS* gene appears to have affected the antifungal activities of the VOCs produced by the strain S-16 (Figure 2).

### 3.3. Determination of the Morphological Characteristics of the Fungal Hyphae Treated with the VOCs from Mutant Strains

**Figure 3:**
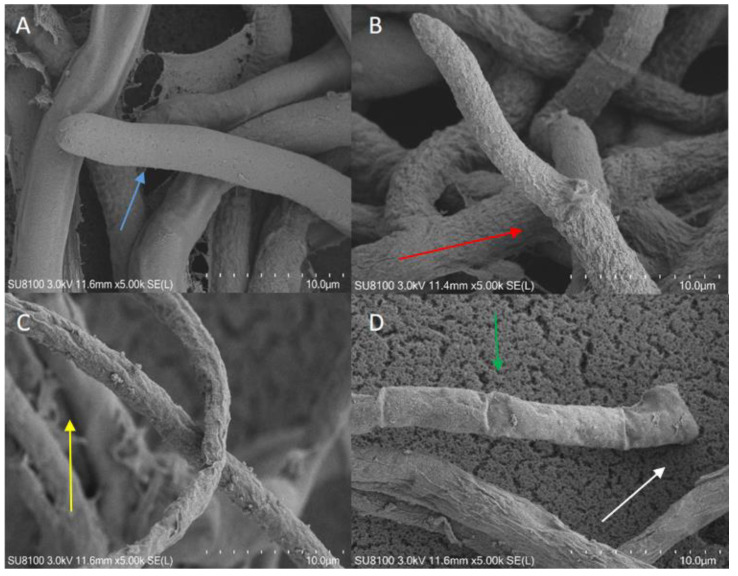
Effects of the volatile compounds from the mutant strains on the hyphal morphology of *Sclerotinia sclerotiorum*. (**A**) Control (blue arrow: smooth hyphae). (**B**)The Δ*thiS* treatment (red arrow: swollen hyphae). (**C**) The Δ*thiS*-c treatment (yellow arrow: jagged hyphae). (**D**) Wild-type S-16 treatment (white arrow: empty segments; green arrow: broken hyphae).

The hyphal structures of *S. sclerotiorum* following treatment with the VOCs from the wild-type and mutant S-16 strains were observed using a scanning-electron microscope. The hyphae of the *S. sclerotiorum* in the untreated control were intact and smooth (Figure 3A), whereas the hyphal structure was extensively damaged by the treatment with the VOCs from the wild-type, Δ*thiS*, and Δ*thiS*-c strains. More specifically, some hyphal expansion was detected (Figure 3B, red arrow). Additionally, the hyphae of the treated mycelia appeared to be relatively flaccid, with uneven surfaces (Figure 3C, yellow arrow). Moreover, empty segments formed (Figure 3D, white arrow), and thin structures or structures with gaps representing a retracted protoplasm were detected (Figure 3D, green arrow). This structural damage may have led to the leakage of cytoplasmic components. Thus, the VOCs produced by the S-16 strain substantially altered the morphology of the hyphae of *S. sclerotiorum* and degraded the cell membrane and wall. However, the mycelial damage caused by the Δ*thiS* treatment was not as extensive as that caused by the Δ*thiS*-c and wild-type S-16 treatments.

### 3.4. Effects of Volatile Compounds from the Mutant Strains on the Formation of Sclerotia

The formation of sclerotia following the treatment with the VOCs from the wild-type and mutant S-16 strains was examined. The untreated *S. sclerotiorum* control produced four sclerotia per Petri dish. In contrast, the *S. sclerotiorum* treated with the wild-type S-16 strain produced only 1.5 sclerotia per Petri dish. The treatment with the Δ*thiS* mutant strain resulted in 3.25 sclerotia per Petri dish. The treatment with the complementary strain produced 1.75 sclerotia per Petri dish. Mutations in *thiS* gene affect the ability of volatiles produced by *B. subtilis* to inhibit sclerotia formation in *S. sclerotiorum* (Table 3).

### 3.5. Test on Detached Sunflower Leaves

**Figure 4:**
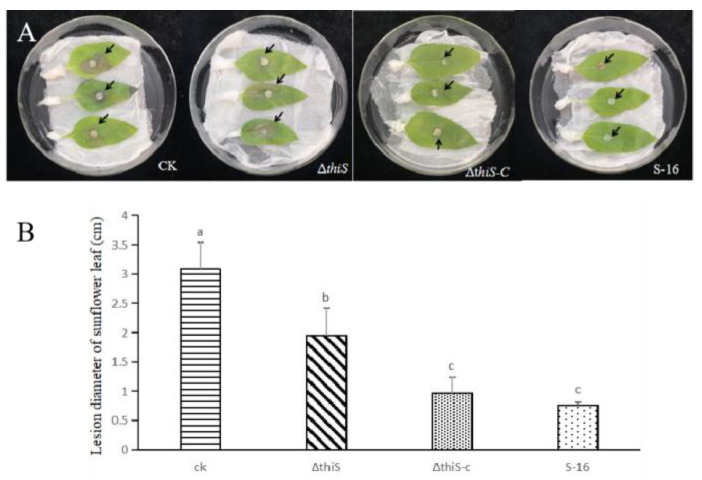
Effects of the volatile compounds from the wild-type S-16, the mutant strain Δ*thiS,* and the complementary strain Δ*thiS*-c on the pathogenicity of *Sclerotinia sclerotiorum*. (**A**) Effect of VOCs produced by wild-type S-16, the mutant strain Δ*thiS*, and the complementary strain Δ*thiS-c* on the development of *S. sclerotiorum* symptoms on sunflower leaves (black arrows indicate the location of the edge of the lesion). (**B**) Lesion areas of sunflower leaves with or without treatment with volatile compounds of wild-type S-16, the mutant strain Δ*thiS*, and the complementary strain Δ*thiS-c*. The data are the mean ± s.d (n = 3). Matching letters on the bars for each column indicate no significant difference according to the LSD test at *p* = 0.05.

The antifungal activities of the VOCs from the wild-type and mutant S-16 strains were compared using detached sunflower leaves. The lesions on the control leaves were relatively large, with diameters of about 3.08 cm. The lesions were smaller on the leaves treated with the VOCs from the wild-type S-16 strain and the derived mutants. After the treatment with the VOCs from the wild-type S-16, the lesion diameters were 0.76 cm, which were smaller than the diameters of the lesions that formed after the treatment with the VOCs from the deletion mutant (1.95 cm). There were no significant differences in the diameters of the *S. sclerotiorum* lesions between the leaves treated with the VOCs from the wild-type and the complementary strains (Figure 4).

### 3.6. Analysis of the 2-MBTH Content in the Mutant Strains

The retention time for the 2-MBTH standard during the gas chromatography–tandem mass spectrometry (GC-MS/MS) analysis was 22.40 min. The contents of 2-MBTH in the wild-type and mutant S-16 strains were compared according to the area of the GC-MS peaks. The peak area was 8,715,422 for the wild-type S-16 strain, but it was only 6,381,940 for the deletion mutant. However, the peak area was partially restored to 7,939,165 for the complementary strain. Thus, these results indicate that the *thiS* gene partly affects the synthesis of 2-MBTH in S-16 (Table 4).

## 4. Discussion

The use of microorganisms and their metabolites is a promising and environmentally friendly alternative to chemical treatments for preventing or controlling plant diseases [30]. A previous study confirmed that the VOCs from the *B. subtilis* S-16 strain have antimicrobial activities that are useful for controlling fungal pathogens [25]. The main VOC from this strain (2-MBTH) can effectively control *Sclerotinia* in sunflowers.

Thiazole-ring-bearing compounds constitute an important class of heterocycles that possess a wide variety of pharmacological activities and have notable pharmaceutical value because of their potential chemotherapeutic properties [31]. The importance of the antimicrobial activity of thiazoles is related to the inclusion of the -S-C=N group as a toxophoric unit in the molecular frame [32]. In addition, benzothiazole has been reported in several studies in terms of its microbiological importance [33,34,35,36]. The potential antimicrobial activity of benzothiazole derivatives was illustrated previously [31,37,38,39,40,41]; thus, the continued search for new antibacterial and antifungal drugs within this group is strongly encouraged. The VOC 2-MBTH is a derivative of benzothiazole. Thiazole moiety 8 is biosynthesized in *B. subtilis* and most of the other bacteria from 1-deoxy-D-xylulose-5-phosphate (1, DXP), glycine, and cysteine in a complex oxidative condensation reaction [42]. This reaction requires five different proteins, namely ThiO, ThiG, *thiS*, ThiF, and a cysteine desulfurase. Glycine oxidase (ThiO) catalyzes the oxidation of glycine to the corresponding imine 7, the sulfur-carrier protein adenylyl transferase (ThiF) catalyzes the adenylation of the carboxy terminus of the sulfur carrier protein (*thiS*-carboxylate), and cysteine desulfurase catalyzes the transfer of sulfur from cysteine to the *thiS*-acyl adenylate to produce *thiS*-thiocarboxylate 6 [41,42,43,44]. Furthermore, ThiG is a thiazole synthase and catalyzes the formation of thiazole from dehydroglycine 7, DXP 1, and *thiS*-thiocarboxylate 6. The early steps of the formation of thiazole have been elucidated [45]. The formation of imine between lysine 96 on ThiG and DXP, followed by tautomerization, produces aminoketone 5, which is then thought to react with *thiS*-thiocarboxylate 6 and dehydroglycine 7 to yield thiazole phosphate 8. The mechanism of the formation of thiazole-phosphate moiety 8 of thiamin in vitro starts with the sulfur-transfer reaction from *thiS*-thiocarboxylate to amino ketone 5, during which a hydroxyl group from 1-deoxy-D-xylulose-5-phosphate (1) (DXP) is transferred to the C-terminal end of the sulfur carrier protein *thiS*-carboxylate [46]. In a previous study, to determine whether *thiS* influenced the synthesis of thiazole in *B. subtilis* S-16, a series of mutant strains was generated. The related gene was functionally verified by comparing the basic biological characteristics of the wild-type and mutant strains.

As expected, compared with the wild-type control, the ∆*thiS* gene knockout strain grew more slowly. In contrast, the growth rate of the ∆*thiS*-c complementary strain was not significantly different from that of the wild-type strain. Hence, *thiS* influences the growth of *B. subtilis* S-16. The growth curves of cultured bacteria can be divided into distinct phases [47]. During the logarithmic growth phase, the bacteria divide vigorously, with a short and stable generation time, resulting in an exponential increase in the number of cells. Thus, the logarithmic phase is often used for microbial fermentation and culturing. During the stationary phase, many microbial metabolites are produced and accumulated. Accordingly, the production of useful metabolites may be enhanced by prolonging the stationary phase [48]. In the current study, the in-frame deletion of *thiS* resulted in delayed growth and the accumulation of secondary metabolites was affected, ultimately leading to weakened antimicrobial activity.

The contents of 2-MBTH in the three bacterial strains were quantitatively analyzed using a GC-MS system. The lack of *thiS* expression affected the production of 2-MBTH. Accordingly, we determined that *thiS* was involved in the synthesis of 2-MBTH in the *B. subtilis*. Furthermore, the volatile compounds produced by the wild-type control, the deletion mutant, and the complementary strain inhibited the mycelial growth and sclerotial formation of the *S. sclerotiorum*. The antimicrobial activities of S-16 may involve additional compounds containing thiazole and its derivatives. Thiazole synthase can catalyze the synthesis of thiazole, which has antibacterial effects. Moreover, thiazole can be converted by certain enzymes to thiazole derivatives with antibacterial activities. Because the thiazoles in S-16 cells flow into metabolic pathways that synthesize antimicrobial substances, the knockdown of thiazole synthase genes can affect antimicrobial activity.

The antimicrobial properties and their effects on the formation of sclerotia varied among the *B. subtilis* strains analyzed. The *S. sclerotiorum* hyphae treated with VOCs were observed using a scanning-electron microscope, which revealed that the VOCs produced by the three examined strains adversely affected the morphological structures of the *S. sclerotiorum* hyphae to a certain extent. The differences in the degree of damage to the mycelial morphology of *S. sclerotiorum* may not have been caused only by the diversity in the VOCs produced by the *B. subtilis* strains. The *S. sclerotiorum* discs treated with the VOCs produced by the three *B. subtilis* strains were used to inoculate fresh and healthy sunflower leaves to clarify the effect of the *thiS* on the *S. sclerotiorum*’s fungal pathogenicity. The results indicated that the deletion of the *thiS* weakened the inhibitory effects of the VOCs against the *S. sclerotiorum*’s fungal pathogenicity. This was in contrast to the considerable decrease in the *S. sclerotiorum*’s fungal pathogenicity following the treatments with the VOCs from the complementary strain and the wild-type control.

The volatiles produced by *B. subtilis* include γ-patchoulene, 3-methylbutanal, 1-octen3-ol, 2-undecanone, 2-nonanone, 3-methylbutanoate, 2-methylbutan-1-ol, 4-methyl-2-heptanone, ethanethioic acid, and dimethyltrisulfide-2,3,6-trimethylphenol. The antifungal activities of several of these chemicals have already been evaluated. These compounds were detected during the analysis of the gaseous volatiles produced by S-16. In this study, the gaseous volatiles produced by the deletion mutant still had antimicrobial effects. These findings may reflect the antimicrobial activities of other gaseous volatile components.

The VOC-based bacteriostatic effects of the deletion mutant differed significantly from those of the wild-type and complementary strains. Therefore, *thiS* is involved in the synthesis of VOCs, which are important for the use of *B. subtilis* S-16 as a biocontrol agent. The results of the current study confirmed the reliability of metabonomic and whole-genome analyses of *B. subtilis* S-16. Furthermore, our results suggest that the *thiS* was involved in the synthesis of 2-MBTH in the *B. subtilis*, which contributed to the biocontrol effects of the latter. The data generated in this study regarding the VOCs of *B. subtilis* S-16 may be useful for enhancing biocontrol measures to protect sunflowers and related species. In the further work, we will continue to study and analyze the inhibitory mechanism through which 2-MBTH inhibits the growth of *S. sclerotiorum* and controls *Sclerotinia* wilt in sunflowers by combining transcriptomics and proteomics. Furthermore, the biological role of *thiS* gene has been extensively investigated in most other bacteria. However, the function of this gene in the synthesis of volatile compounds by *B. subtilis* is unknown. Here, we present the effect of *thiS* on the synthesis of 2-MBTH in *B. subtilis* through knockout-mutant construction and phenotypical characterization. The deletion of the *thiS* gene in *B. subtilis* results in decreased 2-MBTH content and decreased antibacterial activity. The gene-expression-pattern levels of other thiazole synthases may constitute a key regulatory mechanism controlling the synthesis of 2-MBTH in *Bacillus subtilis*. This mechanism is of interest, and we will explore it in our future work.

## 5. Conclusions

The goal of the present study was to determine the impact of the *thiS* gene’s deletion on the 2-MBTH production and antimicrobial activity of *Bacillus subtilis* S-16. We showed that the mutant strain produced less 2-MBTH. The ability of the VOCs produced by the mutant strain to inhibit the growth of the mycelia was also reduced. The production of 2-MBTH and its antimicrobial activities were adversely affected to varying degrees by the deletion of the *thiS.* Accordingly, we determined that the *thiS* was involved in the synthesis of the 2-MBTH in the *B. subtilis*. The functions of other regulatory genes that may interact with thiazole synthesis are yet to be verified, and the idea of modifying *Bacillus subtilis* to make it a more effective biological control agent requires further investigation.

## Figures and Tables

**Table 1:** Tested strains and plasmids used in this experiment.

Strain or Plasmid	Properties and Application	Source or Reference
*B. subtilis*		
*Bacillus subtilis* S-16	Wild-type strain in this study	Kept in our laboratory, isolated from sunflower root.
Δ*thiS*	The *This* gene’s defect mutant, Δ*thiS*	Construct in this study
Δ*thiS*-c	Δ*thiS* supplemented with its native *thiS* gene by allelic exchange	Construct in this study
*E. coli*		
*E. coli* DH5α	Plasmid propagation	Purchased from Takara, Dalian
*plasmid*		
pKSV7	For gene knockout	Purchased from Takara, Dalian
pHY300PLK	For gene knockout	Purchased from Takara, Dalian
pKSV7-*thiS*	For gene knockout, Amp+; cm+	Construct in this study
pHY300PLK-*thiS*	For gene complementation, Cm+	Construct in this study
*Sclerotinia sclerotiorum X-8*	For testing the inhibition of mycelial growth by VOCs	Kept in our laboratory, isolated from sunflower plants.

**Table 2:** The sequence of the primers of mutation in the *thiS* gene.

Gene Name	Primer (5′-3′)	Restriction Sites
*thiS*-1	CGG AAT TCG ATG GAC GAT TTG GAC TCT G	*EcoR*I
*thiS*-2	CGG GGT ACC ATA TCT GAA CCG CCT CCT TG	*Kpn*I
*thiS*-3	CGG GTA CCA GGC GGA TGA GCA TGT TAA C	*Kpn*I
*thiS*-4	CGG GAT CCG ACT GCT TGC CGT TCC GTA C	*BamH*I
*thiS*-promoter-F	CGGAATTCCTGCAAGCCGGTAGAAGAG	*EcoR*I
*thiS*-promoter-R	CGGGATCCGCACGCGGTGAATAGTAGAG	*BamH*I
*thiS*-comF	CGGGATCCAGGCGGTTCAGATATGATGC	*BamH*I
*thiS*-comR	CCCAAGCTTCTCATCCGCCTCCTAC	*Hind*III

**Table 3:** Effects of volatile compounds from the mutant strains on the formation of sclerotia *.

Strain	The Number of Sclerotia (Single-Digit)	Sclerotium Weight (g)
ck	4 ± 0.82 a	0.22 ± 0.06 a
Δ*thiS*	3.25 ± 0.82 a	0.20 ± 0.03 a
Δ*thiS-c*	1.75 ± 0.5 b	0.17 ± 0.02 a
S-16	1.5 ± 0.58 b	0.15 ± 0.07 a

* Data are average area ± the standard deviation of three replicates. Means in the third column of with different letters (a and b) are significant different (*p* < 0.05) according to Duncan’s multiple range tests.

**Table 4:** Analysis of the 2-MBTH contents in the mutant strains.

Active VOCs	Retention Time (Min)	Height	Area	Area (%)	Compound
2-methyl benzothiazole	22.551	36,015,872	3,001,261,211	94.85	2-methyl benzothiazole
S-16	22.394	258,676	8,715,422	0.405	2-methyl benzothiazole
Δ*thiS*	22.400	211,887	6,381,940	0.215	2-methyl benzothiazole
Δ*thiS-c*	22.402	248,132	7,939,165	0.403	2-methyl benzothiazole

## Data Availability

The authors confirm that the data supporting the findings of this study are available within the article.

## References

[1] Jing W., Jianru Z., Chaomin C., Xia C., Zhiyong C., Xiaoshuo L., Xi C., Shuyin W. (2006). Research progress of sunflower *Sclerotinia sclerotiorum*. J. North. Agric..

[2] Bloomfield B.J., Alexander M. (1967). Melanins and Resistance of Fungi to Lysis. J. Bacteriol..

[3] Dean R., Kan J., Pretorius Z.A., Hammond-Kosack K.E., Pietro A.D., Spanu P.D., Rudd J.J., Dickman M., Kahmann R., Ellis J. (2012). The Top 10 fungal pathogens in molecular plant pathology. Mol. Plant Pathol..

[4] Guoqing L., Hongzhang H. (1997). Study on diversity of sclerotia germination of Sclerotia of *Sclerotiorum sclerotiorum*. J. Plant Prot..

[5] Fernando W.G.D., Ramarathnam R., Krishnamoorthy A.S., Savchuk S.C. (2005). Identification and use of potential bacterial organic antifungal volatiles in biocontrol. Soil Biol. Biochem..

[6] Minuto A., Spadaro D., Garibaldi A., Gullino M.L. (2006). Control of soilborne pathogens of tomato using a commercial formulation of *Streptomyces griseoviridis* and solarization. Crop Prot..

[7] Quansheng L., Zongming X., Zheng L., Guoli Z., Dongmei W., Ying T. (2018). Screening and identification of antagonistic bacterium H14 against *Verticillium dahliae* Kleb. and its antagonistic mechanisms. J. Plant Prot..

[8] Afzal S. (2019). Plant beneficial endophytic bacteria: Mechanisms, diversity, host range and genetic determinants. Microbiol. Res..

[9] Arbi A.E., Rochex A., Chataigne G., Bechet M., Lecouturier D., Arnauld S., Gharsallah N., Jacques P. (2016). The Tunisian oasis ecosystem is a source of antagonistic *Bacillus* spp. producing diverse antifungal lipopeptides. Res. Microbiol..

[10] Cochrane S.A., Vederas J.C. (2016). Lipopeptides from Bacillus and *Paenibacillus* spp.: A Gold Mine of Antibiotic Candidates. Med. Res. Rev..

[11] Olishevska S., Nickzad A., Déziel E. (2019). Bacillus and *Paenibacillus* secreted polyketides and peptides involved in controlling human and plant pathogens. Appl. Microbiol. Biotechnol..

[12] Minerdi D., Bossi S., Gullino M.L., Garibaldi A. (2010). Volatile organic compounds: A potential direct long-distance mechanism for antagonistic action of *Fusarium oxysporum* strain MSA 35. Environ. Microbiol..

[13] Asghar H., Mohammad P. (2010). A Review on Biological Control of Fungal Plant Pathogens Using Microbial Antagonists. J. Biol. Sci..

[14] Tyc O., Garbeva P. (2017). Poster OlafTyc FEMS2017 The Ecological Role of Volatile and Soluble Secondary Metabolites produced by Soil Bacteria. Trends Microbiol..

[15] Ryu C.M., Farag M.A., Hu C.H., Reddy M.S., Kloepper J.W. (2003). Bacterial volatiles promote growth in Arabidopsis. Proc. Natl. Acad. Sci. USA.

[16] Breinig S., Schiltz E., Fuchs G. (2000). Genes Involved in Anaerobic Metabolism of Phenol in the Bacterium *Thauera aromatica*. J. Bacteriol..

[17] Zou C.S., Mo M.H., Gu Y.Q., Zhou J.P., Zhang K.Q. (2007). Possible contributions of volatile-producing bacteria to soil fungistasis. Soil Biol. Biochem..

[18] Alström S. (2010). Characteristics of Bacteria from Oilseed Rape in Relation to their Biocontrol Activity against *Verticillium dahliae*. J. Phytopathol..

[19] Koitabashi M. (2005). New biocontrol method for parsley powdery mildew by the antifungal volatiles-producing fungus Kyu-W63. J. Gen. Plant Pathol..

[20] Guevara-Avendano E., Carrillo J.D., Ndinga-Muniania C., Moreno K., Mendez-Bravo A., Guerrero-Analco J.A., Eskalen A., Reverchon F. (2018). Antifungal activity of avocado rhizobacteria against *Fusarium euwallaceae* and *Graphium* spp., associated with *Euwallacea* spp. nr. fornicatus, and *Phytophthora cinnamomi*. Antonie Van Leeuwenhoek.

[21] Qi W. (2014). Cloning of the Bacteriostat Related Gene and Identification of Antifungal Substances from Bacillus Subtilis S-16.

[22] Jinghan H., Dong W., Baozhu D., Huanwen M., Jun Z., Hongyou Z. (2021). The inhibitory effect of the volatile compound 2-methyl benzothiazole on pathogenic fungi *Sclerotinia sclerotiorum* and *Botrytis cinerea*. J. Plant Prot..

[23] Lijun C., Yanan Z., Chunsheng W., Jinzhu C., Yinli J., Yuehua C. (2020). Analysis of the fumigation inhibition mechanism of essential oil from Chenopodium ambrosioides against the *Botrytis cinerea*. J. Plant Prot..

[24] Ginsberg G., Toal B., Kurland T. (2011). Benzothiazole Toxicity Assessment in Support of Synthetic Turf Field Human Health Risk Assessment. J. Toxicol. Environ. Health Part A.

[25] Yiming Z. (2010). Isolation and Identification of a Biocontrol Bacterium against Sclerotinia Sclerotinia of Sunflower and Preliminary Study on Its Antibacterial Mechanism.

[26] Yaguang H. (2016). The Study on the Pathogenicity Differentiation and Sclerotial Formation Mechanism of Sunflower Sclerotinia sclerotiorum.

[27] Smith K., Youngman P. (1992). Use of a new integrational vector to investigate compartment-specific expression of the *Bacillus subtilis* spoIIM gene. Biochimie.

[28] Xu Y.B., Mai C., Ying Z., Wang M., Ying W., Qiu-Bin H., Xue W., Wang G. (2014). The phosphotransferase system gene ptsI in the endophytic bacterium *Bacillus cereus* is required for biofilm formation, colonization, and biocontrol against wheat sharp eyespot. Fems Microbiol. Lett..

[29] Ishiwa H., Shibahara H. (1985). New shuttle vectors for Escherichia coli and Bacillus subtilis. II: Plasmid pHY300PLK, a multipurpose cloning vector with a polylinker, derived from pHY460. Jpn. J. Genet..

[30] Sahni S., Sarma B.K., Singh K.P. (2008). Management of *Sclerotium rolfsii* with integration of non-conventional chemicals, vermicompost and *Pseudomonas syringae*. World J. Microbiol. Biotechnol..

[31] Yurttaş L., Özkay Y., Duran M., Turan-Zitouni G., Özdemir A., Cantürk Z., Küçükoğlu K., Kaplancıklı Z.A. (2016). Synthesis and antimicrobial activity evaluation of new dithiocarbamate derivatives bearing thiazole/benzothiazole rings. Phosphorus Sulfur Silicon Relat. Elem..

[32] Taori K., Paul V.J., Luesch H. (2008). Structure and Activity of Largazole, a Potent Antiproliferative Agent from the Floridian Marine Cyanobacterium *Symploca* sp.. J. Am. Chem. Soc..

[33] Soni B., Ranawat M.S., Sharma R., Bhandari A., Sharma S. (2010). Synthesis and evaluation of some new benzothiazole derivatives as potential antimicrobial agents. Eur. J. Med. Chem..

[34] Sharma P.C., Sinhmar A., Sharma A., Rajak H., Pathak D.P. (2013). Medicinal significance of benzothiazole scaffold: An insight view. J. Enzym. Inhib. Med. Chem..

[35] Franchini C., Muraglia M., Corbo F., Florio M.A., Mola A.D., Rosato A., Matucci R., Nesi M., Bambeke F.V., Vitali C. (2010). Synthesis and biological evaluation of 2-mercapto-1,3-benzothiazole derivatives with potential antimicrobial activity. Archiv. Pharm..

[36] Facchinetti V., Reis R., Gomes C., Vasconcelos T. (2012). Chemistry and biological activities of 1,3-benzothiazoles. Mini-Rev. Org. Chem..

[37] Lad N.P., Manohar Y., Mascarenhas M., Pandit Y.B., Pandit S.S. (2017). Methylsulfonyl benzothiazoles (MSBT) derivatives: Search for new potential antimicrobial and anticancer agents. Bioorganic Med. Chem. Lett..

[38] Padalkar V.S., Borse B.N., Gupta V.D., Phatangare K.R., Patil V.S., Umape P.G., Sekar N. (2016). Synthesis and antimicrobial activity of novel 2-substituted benzimidazole, benzoxazole and benzothiazole derivatives. Arab. J. Chem..

[39] Trapani A., Catalano A., Carocci A., Carrieri A., Mercurio A., Rosato A., Mandracchia D., Tripodo G., Schiavone B.I.P., Franchini C. (2019). Effect of Methyl-β-Cyclodextrin on the antimicrobial activity of a new series of poorly water-soluble benzothiazoles. Carbohydr. Polym..

[40] Al-Talib M., Al-Soud Y.A., Abussaud M., Khshashneh S. (2011). Synthesis and biological evaluation of new benzothiazoles as antimicrobial agents. Arab. J. Chem..

[41] Park J.H., Dorrestein P.C., Zhai H., Kinsland C., Mclafferty F.W., Begley T.P. (2003). Biosynthesis of the Thiazole Moiety of Thiamin Pyrophosphate (Vitamin B1). Biochemistry.

[42] Wang C., Xi J., Begley T.P., Nicholson L.K. (2001). Solution structure of *thiS* and implications for the evolutionary roots of ubiquitin. Nat. Struct. Biol..

[43] Settembre E.C., Dorrestein P.C., Park J., Augustine A., Begley T.P., Ealick S.E. (2003). Structural and mechanistic studies on ThiO, a glycine oxidase essential for thiamin biosynthesis in Bacillus subtilis. Biochemistry.

[44] Lauhon C.T., Kambampati R. (2000). The iscS Gene in Escherichia coli Is Required for the Biosynthesis of 4-Thiouridine, Thiamin, and NAD. J. Biol. Chem..

[45] Dorrestein P.C., Zhai H., Taylor S.V., Mclafferty F.W., Begley T.P. (2004). The biosynthesis of the thiazole phosphate moiety of thiamin (vitamin B1): The early steps catalyzed by thiazole synthase. J. Am. Chem. Soc..

[46] Dorrestein P.C., Zhai H., Mclafferty F.W., Begley T.P. (2004). The Biosynthesis of the Thiazole Phosphate Moiety of Thiamin: The Sulfur Transfer Mediated by the Sulfur Carrier Protein *thiS*. Cell Chem. Biol..

[47] Yanlei Z. (2016). Study on experimental method of measuring bacterial growth curve. J. Microbiol..

[48] Jixian M., Zhigang W., Changhe W. (2012). Comparison of common microbe number determination methods. Biol. Teach..

